# Military Values, Military Virtues, and Vulnerable Narcissism among Cadets of the Swiss Armed Forces—Results of a Cross-Sectional Study

**DOI:** 10.3390/ejihpe14070138

**Published:** 2024-07-19

**Authors:** Immanuel Schkade, Dena Sadeghi-Bahmani, Undine E. Lang, Rebecca K. Blais, Zeno Stanga, Ismail I. Ülgür, Serge Brand, Hubert Annen

**Affiliations:** 1 Psychiatric Hospital of the University of Basel, 4002 Basel, Switzerland; immanuel.schkade@upk.ch (I.S.); undine.lang@upk.ch (U.E.L.); 2 Department of Psychology, Stanford University, Stanford, CA 94305, USA; bahmanid@stanford.edu; 3 Department of Epidemiology and Population Health, Stanford University, Stanford, CA 94305, USA; 4 Psychology Department, Arizona State University, Tempe, AZ 85287, USA; rebecca.blais@asu.edu; 5 Centre of Competence for Military and Disaster Medicine, Swiss Armed Forces, 3008 Bern, Switzerland; zeno-giovanni.stanga@vtg.admin.ch (Z.S.); ismail.uelguer@vtg.admin.ch (I.I.Ü.); 6 Division of Diabetes, Endocrinology, Nutritional Medicine and Metabolism, University Hospital and University of Bern, 3010 Bern, Switzerland; 7 Center of Affective, Stress and Sleep Disorders (ZASS), Psychiatric Clinics of the University of Basel, 4002 Basel, Switzerland; 8 Division of Sport Science and Psychosocial Health, Department of Sport, Exercise, and Health, Department of Medicine, University of Basel, 4052 Basel, Switzerland; 9 Substance Abuse Prevention Research Center, Kermanshah University of Medical Sciences, Kermanshah 6715847141, Iran; 10 Sleep Disorders Research Center, Kermanshah University of Medical Sciences, Kermanshah 6715847141, Iran; 11 School of Medicine, Tehran University of Medical Sciences, Tehran 1416634793, Iran; 12 Center for Disaster Psychiatry and Disaster Psychology, Psychiatric Clinics of the University of Basel, 4002 Basel, Switzerland; 13 Military Academy, Swiss Federal Institute of Technology ETH Zurich, 8903 Birmensdorf, Switzerland; hubert.annen@milak.ethz.ch

**Keywords:** military values, military virtues, organizational citizenship behavior, resilience, vulnerable narcissism

## Abstract

**Background**: For military leaders, military values and virtues are important psychological prerequisites for successful leadership and for ethical and moral military behavior. However, research on predictors of military values and virtues is scarce. Given this background, we investigated whether Organizational Citizenship Behavior (OCB), resilience, and vulnerable narcissism might be favorably or unfavorably associated with military values and virtues, and whether vulnerable narcissism could moderate the association between the OCB-by-resilience-interaction, and military virtues. **Methods**: A total of 214 officer cadets (mean age: 20.75 years; 96.8% males) of the Swiss Armed Forces (SAF) volunteered to take part in this cross-sectional study. They completed a booklet of self-rating scales covering dimensions of military values and military virtues, OCB, resilience, and vulnerable narcissism. **Results**: Higher scores for military virtues were associated with higher scores for military values, OCB, and resilience, and with lower scores for vulnerable narcissism. Multiple regression models showed that higher scores for OCB and resilience were associated with military values and virtues. Vulnerable narcissism moderated the association between military virtues, and the OCB-by-resilience-interaction: the higher the vulnerable narcissism, the more the OCB-by-resilience-interaction was associated with lower scores for military virtues. **Conclusions**: Among cadets of the SAF, the associations between military values, military virtues, OCB, and resilience were highly intertwined, while vulnerable narcissism appeared to attenuate the association between military virtues, OCB, and resilience.

## 1. Introduction

Military leaders are expected to behave by strictly following the ethical and moral standards of warship [1], and to respect the International Laws of Nations, both in times of peace and war [1,2]. Noteworthy, in antiquity, military values and military virtues were critical for military behavior. Indeed, Thucydides used the example of the Peloponnesian War and described the habituation of the Greek public to the “(…) disregard of religious rules and rules similar to those of international law to which one had previously felt obliged in the conduct of war. (…) The peak of inhumanity was reached when the Athenians in the summer of 413 BC, for lack of financial resources, released a bloodthirsty Thracian mercenary force back home and, under the leadership of the Athenian officer Dieitrephes, slaughtered (…) the entire civilian population and, above all, all the children gathered in the school (…)” [3,4]. Shifting from the ancient past to the recent past, in April 2004 the incidents at Abu Ghraib prison became public, where US soldiers carried out inhumane acts on prisoners [5]. Identically, the current examples of war crimes committed by the Russian army describe by the UN Office of the High Commissioner for Human Rights [6] demonstrate that the importance of military values and virtues of military leaders have not lost any of their importance [7,8,9]. On the contrary, similar to the literature from 2500 years ago, the importance of military values and virtues has not diminished. Zanetti et al. [10,11] dealt in depth with military values and virtues and analyzed their psychological structure and related behavior. Military values, such as trust, comradeship or respect, are understood as guiding principles adapted to the specific culture of the military environment [11]. Complementarily, military virtues, such as courage, consideration or modesty, are understood as moral traits [12] adapted to the specific culture of the military environment [11].

### 1.1. Military Values, Military Virtues, and Military-Related Behavior

What is not fully clear yet is what factors give rise to military values and virtues. Current literature [10] showed that higher scores for personal values correlated with higher scores for motivation to lead [13], leader performance [14], and optimal team performance [15]. Other factors that may play a role are Organizational Citizenship Behavior (OCB), resilience and vulnerable narcissism, defined as a person’s tendency to seek attention and admiration.

More specifically, within the area of military psychology, OCB has gained increased attention. Here, OCB is understood as an individual’s behavior that is discretionary, not directly or explicitly recognized by the formal reward system, and that in the aggregate promotes the effective functioning of the organization [16]. Organ [17] redefined OCB as “contributions to the maintenance and enhancement of the social and psychological context that supports task performance (…)” (p. 91).

In the military context, various trust-promoting behaviors of superiors have been favorably associated with the OCB of subordinates [18]. Further, higher levels of OCB predicted voluntariness to pursue a career as militia cadre [19], and students who performed military service exhibited higher scores for OCB than students with no military experience [20].

In search of further possible dimensions associated with personal values, it appeared that resilience might be such a factor [21]. The concept of resilience [22] was initially developed from longitudinal studies with children [23,24,25,26,27], where resilience described a child’s ability to psycho-socially develop favorably despite difficult life and environmental circumstances. More broadly, resilience is understood as the ability of a person to retain mental health despite psychological adversity [28,29,30]. Resilience appears to be a protective factor against stress [31,32] and to favorably influence coping with stress [31,33,34], with adversity [32,35,36,37,38,39] and with traumatic events [40,41].

In the military context, higher scores for resilience were associated with lower scores for chronic stress [42], and cadets’ resilience training appeared to reduce subjectively perceived stress [43]. Brief resilience training seemed to improve the way cadets were dealing with stressful situations and led them to recover more quickly [44]. Further, among cadets, specific interventions to increase resilience led to higher scores for resilience, which in turn were associated with higher scores for military performance [45], task-oriented coping and psychological well-being [28], and with lower scores for perceived and psychological distress [45]. Finally, higher scores for personal values, though not military values and military virtues, were associated with fewer psychological symptoms, such as post-traumatic, depressive, anxious and somatoform symptoms [21,46]. However, so far, no studies have examined the associations between resilience and military values and virtues, and further psychological dimensions.

Therefore, with the dimension of vulnerable narcissism, we investigated another but seemingly opposed factor to those of previous constructs. The American Psychiatric Association DSM-5-TR [47] defines narcissism as a distinct personality disorder, characterized, for instance, by inflated self-esteem, craving for admiration and a lack of empathy, attachment and intimacy. Narcissistic traits can also be on a normal continuum [48,49], and Wink [50] described two types of narcissism: grandiose narcissism, which reflects traits related to grandiosity, aggression, and dominance, and vulnerable narcissism, which is primarily characterized by hypersensitivity to others’ opinions, an intense desire for approval, and by defensiveness [51,52,53].

Vulnerable narcissism appears to be related to all forms of aggression among all ages [54], to perpetration in the context of intimate partner violence [55], anxious attachment [56] and to attachment anxiety [57], increased hostility, anger, and shame in children [58], increased shame in young people [59] and low self-esteem among adults [57]. Moreover, higher scores for vulnerable narcissism were associated with higher scores for emotion dysregulation [60,61,62,63], eating disorder symptoms [64,65,66], problematic social media use [51,67], social anhedonia [68], decreased mental toughness [69,70], and hyper-competitiveness [71].

In the military context, aspects of narcissism, such as a strong sense of ego and a high level of self-esteem, were appreciated in leaders, whereas manipulative behaviors had a detrimental impact on leadership qualities [72]. Vulnerable narcissism appeared to be a strong indicator of anti-social factors, such as aggression [73], and to predispose to negative relationships within the unit [74]. Annen, et al. [75] showed in a study of U.S. and Swiss military officer cadets that those scoring high on vulnerable narcissism were also those scoring low on mental toughness and on sleep quality, while those also had higher scores for perceived stress, and higher Dark Triad traits. Notably, Dark Triad traits reflect personalities containing dimensions of narcissism, Machiavellianism, and psychopathy, which are socially highly discouraged. Given this background, it appears plausible that vulnerable narcissistic traits might unfavorably be associated with military values and military virtues. However, to our understanding, it appears that there is no research investigating this relationship between vulnerable narcissism and military values and virtues.

### 1.2. The Present Study

To gain a deeper understanding of the co-occurring psychological dimensions associated with military values and virtues, we examined their associations with various psychological constructs, such as OCB, resilience, and vulnerable narcissism.

The following three hypotheses and two research questions were formulated. First, following previous research [19,20,76], we expected that military values and virtues and OCB would be positively associated. Second, based on previous findings [21,46], we expected that military values and virtues and resilience would be positively associated. Third, and following others [73,74,75], we expected that higher scores for military values, virtues, OCB, and resilience would be associated with lower scores for vulnerable narcissism. The research questions were: first, which psychological constructs (OCB, resilience, and vulnerable narcissism) were statistically more associated with military values and virtues? Second, did vulnerable narcissism moderate the associations between OCB, resilience, and military virtues?

To test the hypotheses and to answer the research questions, cadets of the Swiss Armed Forces were cross-sectionally assessed (see details below). The study should allow the shedding of more light on the nature and predictors of military values and military virtues, which in former studies were associated with leader performance [14], optimal team performance [15] and resilience [21,46].

## 2. Method

### 2.1. Procedures

Cadets of the Swiss Armed Forces were approached during their officer training course. They were fully informed about the aims of the study, and the confidential and anonymous data handling. Further, participation was voluntary, and participation, or non-participation, was neither recorded on the cadets’ military records, nor considered for their future academic career. Thereafter, participants signed the written informed consent and completed a booklet of self-rating questionnaires on socio-demographic information, military values and military virtues, OCB, resilience, and vulnerable narcissism (see details below). Participants needed between 30 and 45 min to complete the booklet. The Military Review Board of the Training and Education Command of the Swiss Armed Forces approved the study, which was conducted in accordance with the seventh and current revision [77] of the Declaration of Helsinki.

### 2.2. Participants

The sample consisted of 214 cadets (all cadets of the officer school) of the Swiss Armed Forces (age: M = 20.75 years; SD = 2.021; range: 18–37 years, 3.2% women, 96.8% men) during their officer training course in spring 2015. Inclusion criteria were: 1. Age 18 years and older; 2. Being cadet and currently attending the officer training course. 3. Willing and able to comply with the study conditions in German. 4. Signed written informed consent. Exclusion criteria: 1. Resign from the study. 2. Leaving the officer training course.

### 2.3. Measures

#### 2.3.1. Socio-Demographic Information

Participants reported on their age (years) and sex at birth (female, male).

#### 2.3.2. Military Values and Virtues

To assess military values and virtues, participants completed the Military Values and Virtues Questionnaire [11]. The self-rating questionnaire consists of 25 items on military values and 42 items on military virtues. Typical items are: Military values: “Obedience” or “Honesty”; military virtues: “Courage” or “Discipline”. Answers are given on a 4-point Likert scales with the following anchor points: 1 (=values: I do not orient myself by; virtues: does not apply to me at all) to 4 (=values: I do orient myself at any rate by; virtues: does fully apply to me) to rate each of the values as to how far the participant orients as a member of the Armed Forces by those values in everyday military decisions and actions, and to rate each of the virtues in how far the characteristic applies to the participant as a member of the Armed Forces. Higher sum scores reflect more pronounced military values or virtues.

#### 2.3.3. Organizational Citizenship Behavior (OCB)

To assess organizational citizenship behavior, participants completed the German version of the Organizational Citizenship Behavior Questionnaire [78], which was modified for the military context. The self-rating questionnaire consists of 25 items. Typical items are: Altruism: “I help my comrades when they are overloaded with a task”; Conscientiousness: “I am always on time”; Sportsmanship (inverse): “I consume a lot of time complaining about trivial matters”; Courtesy: “I take steps to avoid problems with comrades”; Civil virtue: “I keep abreast of changes in the Armed Forces”. Answers are given on 7-point-Likert scales ranging from 1 (=strongly disagree) to 7 (=strongly agree), with a higher overall sum scores reflecting a more pronounced OCB (Cronbach’s α = 0.81).

#### 2.3.4. Resilience

To assess the grade of resilience, participants completed the German version [79] of the Resilience Questionnaire (RS-14) [80,81,82]. The self-rating questionnaire consists of 14 items assessing the grade of resilience, i.e., the psychological strength and ability to cope with challenges, stress and setbacks. Typical items are: “When I’m in a difficult situation, I can usually find my way out of it”, or “My belief in myself gets me through hard times”. Answers are given on 7-point Likert scales with the following anchor points: 1 (=strongly disagree) to 7 (=strongly agree). Higher sum scores reflect more pronounced resilience (Cronbach’s α = 0.89).

#### 2.3.5. The Hypersensitive Narcissism Scale

To assess vulnerable narcissism, participants completed the German version of the Hypersensitive Narcissism Scale (HSNS) [83]. The self-rating questionnaire consists of 10 items. Typical items are: “I am secretly “put out” or annoyed when other people come to me with their troubles, asking me for my time and sympathy”, or “I often interpret the remarks of others in a personal way”. Answers are given on 5-point Likert scales ranging from 1 (=very uncharacteristic or untrue, strongly disagree) to 5 (=very characteristic or true, strongly agree), with higher sum scores reflecting a higher vulnerable narcissistic trait (Cronbach’s α = 0.82).

#### 2.3.6. Analytical Plan

Associations between military values, military virtues, OCB, resilience, and vulnerable narcissism were calculated with a series of Pearson’s correlations. To run multiple regression models, preliminary conditions were met [84,85,86]: N = 214 > 100; predictors explained sufficiently the dependent variables (military values: R = 0.457; R^2^ = 0.209; military virtues: R = 0.667; R^2^ = 0.447); the number of predictors: 3; 3 × 10 = 30 < 214; Durbin-Watson coefficients were (military values: 1.838; military virtues: 1.962), indicating that the residuals of the predictors were independent. Further, the variance inflation factors (VIF) were between 1.425 and 1.625, while there are no strict cut-off points to report the risk of multi-collinearity, VIF < 1 and VIF > 10 indicate multi-collinearity [85,86]. We further explored the resilience x OCB-interaction effects, and the moderating effect of the degree of vulnerable narcissism.

The level of significance was set at α < 0.05. All statistical calculations were performed with SPSS^®^ 29.0 (IBM Corporation, Armonk, NY, USA) for Apple Mac^®^.

## 3. Results

### 3.1. General Socio-Demographic Information

A total of 214 cadets took part in the study. Their mean age was 20.75 years (SD = 2.03). Of these, 207 (=96.8%) were males, and seven (3.8%) were females.

### 3.2. Associations between Military Values, Military Virtues, Organizational Citizenship Behavior, Resilience, and Vulnerable Narcissism

Table 1 reports the descriptive statistical indices and the correlation coefficients (Pearson’s correlations) for military values, military virtues, OCB, resilience, and vulnerable narcissism. Please note that correlation coefficients are fully reported in the Table, and accordingly not repeated in the text.

Higher scores for military values were associated with higher scores for military virtues, OCB, and resilience, and with lower scores for vulnerable narcissism (non-significant correlation coefficient).

Higher scores for military virtues were associated with higher scores for OCB and resilience, and with lower scores for vulnerable narcissism.

Higher scores for OCB were associated with higher scores for resilience and with lower scores for vulnerable narcissism.

Higher scores for resilience were associated with lower scores for vulnerable narcissism.

Higher scores for vulnerable narcissism were associated with lower scores for OCB.

Overall, the pattern was such that higher military values, higher military virtues, higher scores for OCB, and resilience were intercorrelated, while these scores, except for military values, were associated with lower scores for vulnerable narcissism.

### 3.3. Psychological Dimensions Associated with Military Values

To further statistically associate military values with OCB, resilience, and vulnerable narcissism, a multiple regression analysis was performed.

Table 2 reports the equation model. Higher scores for OCB and resilience were statistically associated with military values, while vulnerable narcissism was excluded from the equation, as it did not reach statistical significance.

### 3.4. Psychological Dimensions Associated with Military Virtues

To further statistically associate military virtues with OCB, resilience, and vulnerable narcissism, a multiple regression analysis was performed.

Table 3 reports the equation model. Higher scores for OCB and resilience were statistically associated with military values, while the following dimensions were excluded from the equation, as they did not reach statistical significance: vulnerable narcissism; resilience × OCB-interaction; resilience × OCB × vulnerable narcissism-interaction.

### 3.5. Associations between Military Virtues, Organizational Citizenship Behavior, Resilience, and Vulnerable Narcissism

In a further step we explored the complex associations between military virtues, OCB, resilience, and vulnerable narcissism. The observations were as follows:

The regression model in Table 3 shows that higher scores for OCB and resilience were positively associated with military virtues. However, the multiplication of the residuals of OCB and resilience was not associated with military virtues (R= −0.07). This zero-association was counter-intuitive, and accordingly, we further explored whether vulnerable narcissism might have moderated such an association. To this end, vulnerable narcissism scores (mean = 2.70; SD = 0.46) were split into three categories: Low vulnerable narcissism (n = 66; m = 2.20; SD = 2.24), medium vulnerable narcissism (n = 78; m = 2.71; SD = 0.08) and high vulnerable narcissism (n = 70; m = 3.20; SD = 0.26).

**Figure 1:**
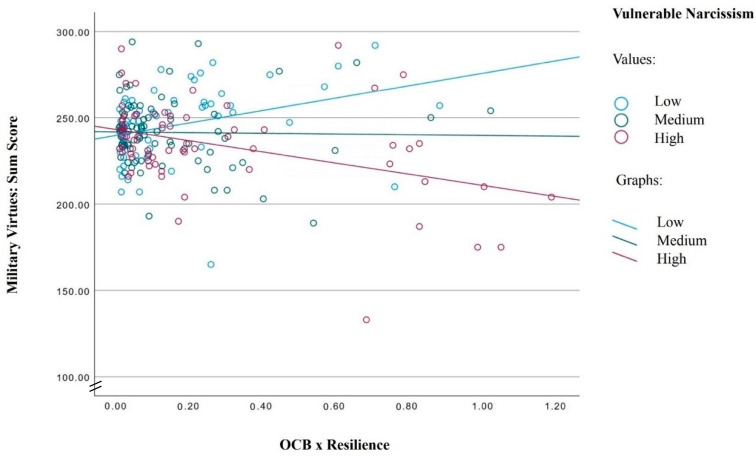
Scatter plot beween the OCB × Resilience-interaction and Military Virtues, separated by low, medium and high Vulnerable narcissim categories.

Correlation coefficients were as follows:

The overall correlation coefficient between military virtues and the multiplication of OCB and resilience was r = −0.15; the correlation coefficient was r = 0.23 among those with low vulnerable narcissism; r = 0.03 among those with medium vulnerable narcissism, and r = −0.33 among those with high vulnerable narcissism. These complex associations are shown also in Figure 1.

To summarize, the complex association between military virtues, OCB and resilience was moderated by vulnerable narcissism categories. Simply put, the higher the vulnerable narcissism, the more decreased the association between military virtues and the OCB–resilience-link.

## 4. Discussion

The aims of the present study were to explore the associations between military values, military virtues, Organizational Citizenship Behavior (OCB), resilience, and vulnerable narcissism among cadets of the Swiss Armed Forces (SAF). The key results were that higher scores for military values, military virtues, OCB, and resilience were associated with each other, while negative associations for these scores were observed for vulnerable narcissism. Further, higher scores for OCB and resilience were statistically associated with higher scores for military values and virtues. Importantly, the associations between military virtues, and the OCB–resilience-link were moderated by the categories of low, medium, and high vulnerable narcissism. The present findings expand upon the current literature in the following three ways. First, we showed that OCB, resilience, and vulnerable narcissism were associated with military values and virtues, though in a complex fashion. Second, more specifically, vulnerable narcissism trait scores might be considered a problematic factor for military virtues. Third, at a practical level, military values and virtues might be further enhanced via resilience, given that specific, well-established, standardized and military-oriented resilience training programs are available.

We formulated three hypotheses and two research questions, which we discuss below as follows.

With the first hypothesis, we assumed that military values and OCB were positively associated, and data did confirm this. Accordingly, the present results mirror previous findings [19,20,76]. However, the present results expand upon previous findings in the following ways. The present pattern of results was observed among cadets of the Swiss Armed Forces (SAF), that is to say, among future officers of the SAF, who are not going to become professional and full-time military officers. More specifically, given the peculiarities of the organization of the SAF, cadets fulfil step by step their military career (militia), and, alongside, they continue their private training or profession. Given this specific context, though not deducible with the present data, it appears conceivable that cadets might also transfer their mindset of military values and virtues, including OCB, to their private and professional life context.

With the second hypothesis, we assumed that military values and virtues, and resilience were positively associated, and data did confirm this. Accordingly, the present results mirror previous findings [21,46]. However, the present results expand upon previous results in that we closed the time gap for the military values–military virtues–resilience-link-research, which appeared to come to a halt about ten years ago [21,46]. As such, we consider the present results as a valuable and timely update in the field of resilience research [42], in general, and in the field of the resilience–military values-link, more specifically.

With the third hypothesis, we expected that higher scores for military values, virtues, OCB, and resilience were associated with lower scores for vulnerable narcissism and, again, data did confirm this. Accordingly, the present results matched what has been observed elsewhere [73,74,75] (for a more comprehensive discussion of this result, see below).

With the first research question we asked about the statistical predictors of military values and virtues, and both OCB and resilience were identified, while vulnerable narcissism was not. The first two results are in line and expand current literature, while the third is not. To explain this pattern of results, we propose the following explanations:

First, as various measures of OCB contain items that are based on ethical convictions [87], it appears plausible that OCB predicts military values and virtues.

Second, resilience correlates significantly with personal values such as, e.g., tradition or universalism [21,46], which overlap with certain military values und virtues. Low resilience could make it more difficult to implement military values and virtues, which in turn could lead to emotional dissonance. Though highly speculative, we hypothesize that people with low resilience could subsequently devalue the orientation to military values and virtues to reduce the inner conflict.

Third, we found that vulnerable narcissism was not statistically significantly negatively associated with military values and military virtues. This result is surprising in that the behavior of military cadre scoring high on vulnerable narcissism might become problematic, as such military cadres appeared to be more aggressive and less thoughtful amongst others [73,74,88].

In our second research question, we examined the aforementioned finding in more depth by exploring dimensions of vulnerable narcissism as a moderator. To our understanding, this is the first investigation of this kind in the field of military research. Importantly, the degree of vulnerable narcissism from low to high, and thus speculatively from low to more problematic (psychopathological) intensity, moderated the associations between the resilience–OCB–military virtues-link. However, the present results are purely statistical results, which are detached from a cadet’s behavior and military performance, above all as regards leadership quality. In this context, previous research showed that military ranking officers scoring high on (vulnerable) narcissism might show manipulative behaviors with detrimental impact on leadership qualities [72]. As mentioned above, vulnerable narcissism appeared to be a strong indicator of antisocial factors, such as aggression [73], and to predispose to negative relationships within the unit [74]. According to Annen et al. [75], U.S. and Swiss military cadres scoring high on vulnerable narcissism also reported higher Dark Triad traits, lower mental toughness, poor sleep quality, and higher scores for perceived stress. Overall, the present pattern of results appears to indicate that dimensions of vulnerable narcissism might moderate the military values–virtues–resilience-link.

### Limitations and Future Directions

Despite the novelty of the results, the following limitations are considered. First, by nature, cross-sectionally collected data do not allow any direction of causality. Accordingly, conclusions drawn from regression models are always statistical, but not causal conclusions. Second, the sample consisted virtually of only male participants. While the prevalence rate of females in the present sample (3.2%) is higher compared to the overall prevalence rates of females within the SAF, ranging between 0.6% [89] and 0.9% [90], the overall results of the present study should not be generalized to all females serving in the SAF. Relatedly, participants of just one cadet military training school were assessed, and such an approach might additionally bias the pattern of results. Third, it is conceivable that further latent and unassessed factors, such as current symptoms of physiological or psychological ill-being, might have biased two or more variables in the same or opposite directions. Specifically, it appeared counter-intuitive and surprising that military values and military virtues were statistically unrelated to narcissism traits. To counter such an apparent contradiction, in the future, semi-structured and qualitative interviews might enable a deepened understanding of unexplored cognitive–emotional processes. Further, given that the present data were gathered in spring 2015, it would be interesting to know, whether and to what extent the pattern of results would be identical or not in the current period of time. In this view, fourth, in future studies, above all, the quantity and quality of sleep should be considered as interfering factor: indeed, there is sufficient evidence that sleep patterns are impaired among military personnel [91,92,93] and, almost by nature, poor sleep and impaired cognitive and emotional processing are intertwined [94,95,96,97]. Fifth, it would have been informative to know whether and, if so, to what extent the current dimensions of military values, military virtues, OCB, resilience, and vulnerable narcissism were related to a cadet’s military qualifications, including their leadership qualifications. Future studies should therefore assess dimensions of military values and virtues longitudinally and associate these dimensions with physiological and psychological well-being, including leadership qualifications. Specifically, it may be interesting to investigate, whether military values and military virtues may change in the course of the officer school in relation to the strenuous physical and psychological pressure and possible selection to continue or to drop out from the officer school.

## 5. Conclusions

Among cadets of the SAF, OCB, resilience, and low vulnerable narcissism were associated and contributing psychological factors of military values and virtues. Traits of vulnerable narcissism might be considered a risk factor for a cadet’s military behavior. Given that standardized and military-tailored resilience training programs are available, such specific interventions might further improve military values and virtues, and dimensions of OCB, while possibly decreasing vulnerable narcissism traits in parallel.

## Figures and Tables

**Table 1:** Descriptive statistical indices and correlation coefficients (Pearson’s correlations) between scores for military values, military virtues, Organizational Citizenship Behavior (OCB), resilience, and vulnerable narcissism.

Dimensions						
	Military Values	Military Virtues	OCB	Resilience	Vulnerable Narcissism	Mean (SD)
Military values	-	0.71 ***	0.40 ***	0.40 ***	−0.12	5.98 (0.54)
Military virtues		-	0.63 ***	0.53 ***	−0.15	5.73 (0.54)
OCB			-	0.55 ***	−0.41 ***	5.90 (0.51)
Resilience				-	−0.23 ***	3.60 (0.36)
Vulnerable narcissism					-	2.72 (0.46)

Note: *** = *p* < 0.001.

**Table 2:** Multiple linear regression with military values as outcome variable, and OCB and resilience as predictors.

Dimension	Variables	Coefficient	Standard Error	Coefficient β	t	*p*	R	R^2^	Durbin-Watson	VIF
Military values	Intercept	58.982	14.878	-	3.963	<0.001	0.457	0.209	1.838	
	OCB	8.41	2.319	0.284	3.627	<0.001				1.625
	Resilience	0.484	0.139	0.255	3.486	<0.001				1.425

Excluded variable: Vulnerable narcissism; t < 1.0; *p* > 0.368.

**Table 3:** Multiple linear regression with military virtues as outcome variable, and OCB and resilience as predictors.

Dimension	Variables	Coefficient	Standard Error	Coefficient β	t	*p*	R	R^2^	Durbin-Watson	VIF
Military virtues	Intercept	41.88	20.907	-	2	0.046	0.667	0.445	1.962	
	OCB	23.705	3.258	0.477	7.275	<0.001				1.625
	Resilience	0.855	0.196	0.268	4.372	<0.001				1.425

Excluded variables: Vulnerable narcissism; resilience × OCB-interaction; resilience × OCB × vulnerable narcissism-interaction: t < 1.58; *p* > 0.114.

## Data Availability

Data belong to the Swiss Armed Forces (SA), and are not shared so far with third parties.
